# Pediatric intracranial high-grade solitary fibrous tumor/hemangiopericytoma with drop metastasis to cervicodorsal spine: a rare case report

**DOI:** 10.1097/RC9.0000000000000224

**Published:** 2026-02-16

**Authors:** Muhammad Hassaan Javaid, Shahid Shah, Gohar Javed, Muddassir Khalid, Rimsha Rani Mushtaq, Iqra Mahmood

**Affiliations:** a Department of Medicine, Shifa College of Medicine, Islamabad, Pakistan; b Department of Neurosurgery, Shifa International Hospital, Islamabad, Pakistan; c Department of Neurosurgery, Nishtar Medical University, Multan, Pakistan; d Department of Pathology, Shifa International Hospital, Islamabad, Pakistan

**Keywords:** central nervous system, drop metastasis, high-grade solitary fibrous tumor, pediatric hemangiopericytoma, postoperative complications

## Abstract

**Background:**

Intracranial solitary fibrous tumor/hemangiopericytoma (SFT/HPC) is extremely rare in children, accounting for fewer than 10% of all SFT/HPCs and significantly fewer primary central nervous system tumors. Pediatric SFT/HPCs of high-grade are particularly aggressive, having a high frequency of recurrence and late metastasis, though spinal “drop” metastases remain, nevertheless, very rare.

**Case Presentation:**

We discuss the case of an 11-year-old girl patient with a 2-year history of recurrent generalized tonic-clonic seizures, worsening headache, vomiting, and acute-onset bilateral visual loss. MRI showed a large frontoparietal mass on the right side, for which neuronavigation-guided craniotomy with gross total resection was performed. Histopathological examination revealed a high-grade (Grade III) SFT/HPC with a high Ki-67 index and characteristic “staghorn” vascular pattern. Postoperatively, the child developed a pseudomeningocele, which was subsequently followed by new neurological deficits. MRI of the cervicothoracic spine revealed a posterior epidural lesion at C7–T1, indicative of early drop metastasis. Referral for further surgery and oncology management was advised, although treatment was refused due to cost.

**Clinical Discussion:**

Pediatric intracranial SFT/HPCs are very uncommon, and such instances have usually been described in isolation. Metastases have a tendency to occur many years following initial treatment, making early spinal dissemination within our patient a rare and ominous occurrence. This case also illustrates pseudomeningocele as a complication, thus highlighting the significance of close follow-up and employment of individualized therapy protocols for postoperative management in children with high-grade disease.

**Conclusion:**

This case points out the virulent biological behavior of pediatric intracranial SFT/HPC and the propensity for early postoperative complications, including unusual early drop metastases. It stresses the need for close monitoring, personalized multidisciplinary management, and early consideration of new neurologic symptoms in children with high-grade intracranial neoplasms. Our publication adds to the limited pediatric literature and highlights the therapeutic challenge faced in resource-poor environments.

## Introduction

Solitary fibrous tumor/hemangiopericytoma (SFT/HPC) is a rare fibroblastic or pericytic mesenchymal neoplasm that can occur in almost any anatomical location^[^1^]^. The identification of the NAB2–STAT6 fusion gene validated the common molecular etiology of these tumors, and the 2016 WHO classification combined them as a single entity with three histologic grades^[^2^]^. Grade I tumors are like classical SFTs with low cellularity, Grade II tumors present hypercellularity with the typical “staghorn” vasculature, and Grade III tumors, formerly known as anaplastic HPCs, are characterized by high mitotic activity (≥5 mitoses per 10 high-power fields)^[^2^]^.>

HIGHLIGHTS

- Pediatric intracranial SFT/HPC is extremely rare.
- High-grade SFT/HPCs exhibit aggressive biology with high recurrence and metastatic potential.
- Our 11-year-old patient developed early postoperative cervicodorsal spinal drop metastasis.
- Pseudomeningocele occurred as an unusual complication after gross total resection.
- This case underscores the need for vigilant follow-up and individualized pediatric management

The 2021 WHO Classification of CNS Tumors continues to group SFT and HPC as a single entity under mesenchymal, non-meningothelial tumors, graded from WHO Grades 1–3 based on mitotic activity and histologic atypia. The hallmark diagnostic feature is STAT6 nuclear immunoreactivity, reflecting the NAB2–STAT6 fusion gene. In children, these tumors are exceedingly uncommon, with fewer than 10% of all SFT/HPCs arising in the pediatric population. Their biological behavior in pediatric patients is poorly understood, and aggressive tendencies may differ significantly from adult tumors^[^3^]^.

According to epidemiological data, the incidence rate is 3.77 per 10 million for SFT/HPC and intracranial lesions account for a mere 0.22% of all primary brain tumors^[^4^]^. Incidence in children is especially low, representing less than 10% of all SFT/HPCs, and intracranial tumors in children are very rarely reported^[^5^]^. Management includes surgical resection with gross total resection, giving the optimum results. Adjuvant radiotherapy should be used in subtotal resection or high-grade pathology, while stereotactic radiosurgery can be applied to recurrence or residual tumor^[^6^]^. SFT/HPCs are even characterized by recurrence and late metastasis, even with aggressive multimodal therapy. Bones, liver, and lungs are the most common metastatic sites, but the occurrence of spinal “drop” metastases following intracranial primaries is very rare, usually years after initial management^[^7,8^]^.

We report a very rare case of pediatric intracranial SFT/HPC with high-grade and unusual postoperative complications and possible drop metastasis to the cervicodorsal spine, which is extremely rare. Our case serves well in describing information concerning the intracranial SFT/HPC diagnostic range, histopathological character, and therapeutic dilemma. Our case adds to the little literature for this uncommon tumor.

“This case report has been reported in line with the SCARE checklist [Kerwan A, Al-Jabir A, Mathew G, Sohrabi C, Rashid R, Franchi T, Nicola M, Agha M, Agha RA. Revised Surgical CAse REport (SCARE) guideline: An update for the age of Artificial Intelligence. Premier Journal of Science 2025:10;100079].”^[^9^]^

The timeline of this case is shown in Table 1.

**Table 1:** Timeline of the case.

Time point	Clinical event
2 years before presentation to hospital	Onset of generalized nocturnal tonic-clonic seizures
6 months prior to presentation	Vomiting before seizures and intermittent right retroorbital headache
1 week before presentation	Acute bilateral vision loss, worsening headaches and vomiting
Upon presentation to hospital	MRI was performed, which showed a right frontoparietal mass
Next 2–3 days	Neuro navigation guided craniotomy with gross total examination
First postoperative week	Development of pseudomeningocele with new neurological deficits
Second postoperative week	Postoperative MRI spine showed cervicodorsal epidural mass consistent with drop metastasis
Third postoperative week	Surgical re-excision and with oncology referral was advised but the patient’s family declined due to financial constraints
Follow-up	Limited due to loss of follow-up and the last known status of patient was that his neurological symptoms persisted with no further intervention

## Case presentation

### Initial presentation

An 11-year-old female presented with a 2-year history of recurrent generalized tonic-clonic seizures, predominantly occurring during sleep, accompanied by uprolling of eyes, frothing from the mouth, and urinary incontinence. Over the past 6 months, she experienced recurrent episodes of nausea and vomiting prior to seizures, along with intermittent right retroorbital headache lasting 1–2 days after seizures, as shown in Table 1.

She developed acute bilateral loss of vision, limiting her to the perception of hand movements. One week later, she experienced a persistent headache and vomiting. There was no history of fever, limb weakness, speech abnormality, or sphincter disturbance. Her past medical history was unremarkable. She was on Sodium Valproate (Epival) 250 mg twice a day for seizure control. She had been placed on dexamethasone 5 days previously for the relief of symptoms.

### Neurological examination

The neurologic examination showed an alert patient with a GCS of 15/15. Speech and comprehension were intact. Cranial nerve examination showed that pupils were bilaterally dilated but reactive, with preserved extraocular movements. Visual acuity was limited to hand movement perception bilaterally. There was no nystagmus, and the tongue and uvula were central. Motor examination showed normal tone, bulk, and power (5/5) in all limbs. Deep tendon reflexes were grade 1, and sensations were intact. Her gait was preserved, although the patient required assistance due to reduced vision.

### Imaging

**Figure 1.**
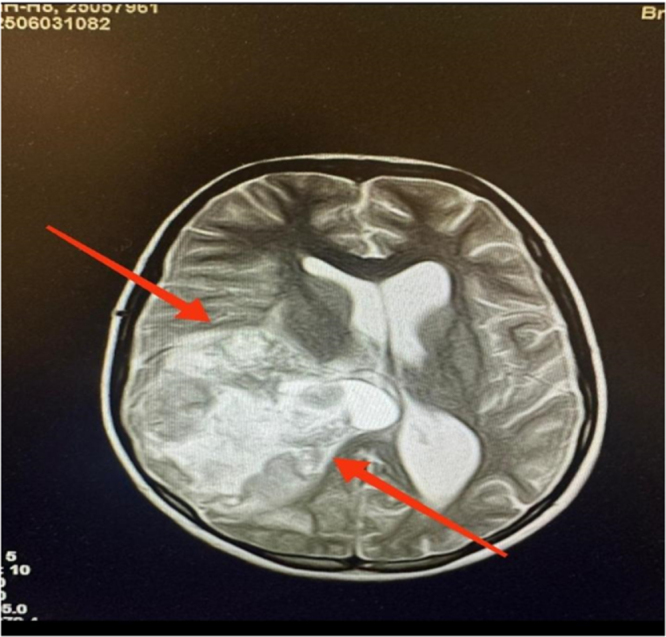
Preoperative contrast-enhanced T1-weighted MRI showing a large, lobulated, intensely enhancing mass in the right frontoparietal region with surrounding vasogenic edema and mass effect.

An electroencephalogram was performed, which showed abnormal, sharp, slow wave discharges across multiple lobes. Magnetic resonance imaging (MRI) of the brain with contrast revealed a large intracerebral space-occupying lesion in the right frontoparietal region, suggestive of a high-grade neoplasm (as shown in Fig. 1). Laboratory investigations were notable for positive Brucella IgG and IgM.

### Diagnosis

For making a diagnosis, the following differentials were considered because of the patient’s symptoms (seizures, raised ICP features, acute visual loss) and radiologic findings: high-grade glioma (e.g., glioblastoma) due to a large infiltrative mass and edema and atypical meningioma (WHO Grade II/III) due to a common extra-axial mass in this region and hypervascular appearance. Primary CNS sarcoma (e.g., embryonal or spindle-cell sarcoma) as well as ependymoma or PNET were considered due to pediatric age and aggressive imaging features. SFT/HPC was suspected due to intense enhancement and vascular “staghorn” pattern.

The diagnosis was made on a combination of clinical, radiologic, and histopathological features: MRI features showed a lesion with strong enhancement, lobulated contours, and prominent flow voids, which raised suspicion for a hypervascular neoplasm rather than a glial tumor. Histopathology features showed the presence of spindle-cell proliferation, high mitotic index, and staghorn-type vasculature, strongly suggesting a HPC-type morphology. According to immunohistochemistry, negative staining for GFAP, IDH, and Olig-2 ruled out glial tumors and retained INI1-excluded rhabdoid tumors. The high Ki-67 index supported high-grade pathology. STAT6 nuclear positivity is the diagnostic hallmark correlating with the NAB2–STAT6 gene fusion, which confirms SFT/HPC.

## Management

### Surgical findings

**Figure 2.**
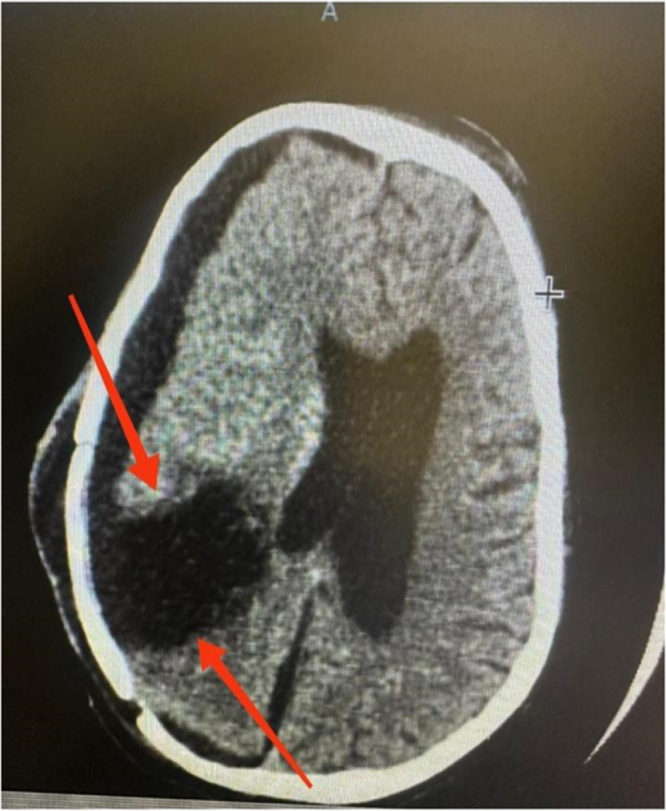
Postoperative MRI demonstrating gross total resection of the intracranial lesion with postoperative changes in the surrounding parenchyma.

The patient was subsequently operated on following neuronavigation-assisted right frontoparietal craniotomy with gross excision of the lesion (as shown in Fig. 4). A histopathological and immunohistochemical examination of the resected tumor specimen showed a well-circumscribed tumor mass with measurements 8.0 × 6.0 × 5.5 cm, including firm greyish cut surfaces with focal cystic degeneration (as shown in Fig. 2).

**Figure 3.**
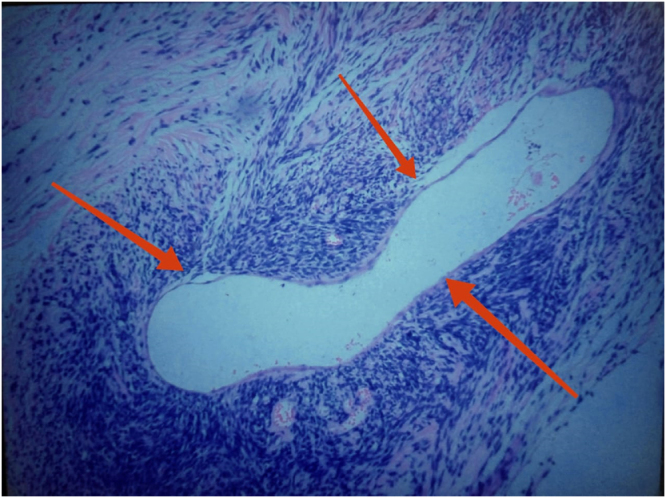
Hematoxylin and eosin stain showing spindle-shaped tumor cells arranged around branching “staghorn” vascular channels, characteristic of SFT/HPC.

### Microscopic findings

Microscopically, there was a spindle cell neoplasm with pleomorphic cells with a high nucleus/cytoplasm ratio, hyperchromatic nuclei, brisk mitotic figures, foci of hyalinization, and prominent staghorn-type blood vessels (as shown in Fig. 3).

**Figure 4.**
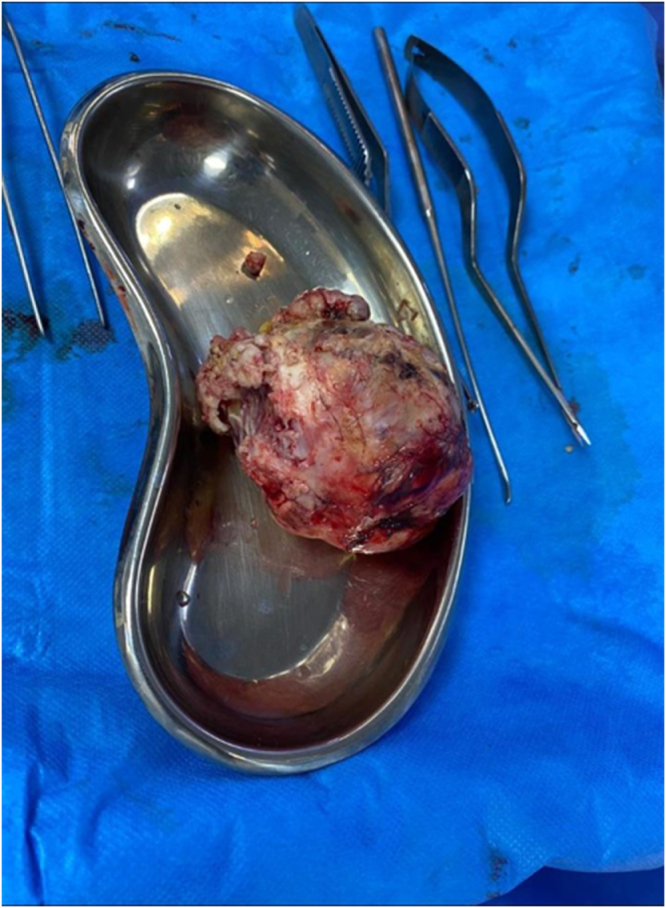
Gross specimen showing an 8 × 6 × 5.5 cm well-circumscribed, firm, gray-white tumor with focal cystic areas.

### Immunohistochemistry findings

Immunohistochemistry revealed that the tumor cells were negative for GFAP, IDH, Olig2, Desmin, EMA, S100, and Synaptophysin, while nuclear expression of INI1 was retained. The Ki-67 labelling index was high, indicating a markedly increased proliferative activity.

These findings confirmed a malignant spindle cell neoplasm, intracranial SFT/HPC of the right temporoparietal region.

In addition to the features described, the tumor demonstrated diffuse CD34 positivity and strong nuclear STAT6 staining, confirming the diagnosis of SFT/HPC. The Ki-67 proliferation index measured approximately 25–30%, consistent with high-grade (WHO Grade III) histology. Areas of geographic necrosis and brisk mitotic activity (>5 mitoses per 10 HPF) further supported an aggressive tumor phenotype. Together, these findings fulfill the diagnostic criteria for WHO Grade III SFT/HPC.

### Postoperative course

Two weeks postoperatively, the patient presented again with scalp swelling, bilateral lower limb weakness, and headache. Evaluation revealed a pseudomeningocele, from which cerebrospinal fluid (CSF) was aspirated.

So the time interval between the occurrence of primary tumor and the drop metastasis was 2 weeks.

### Recurrence/metastasis

**Figure 5.**
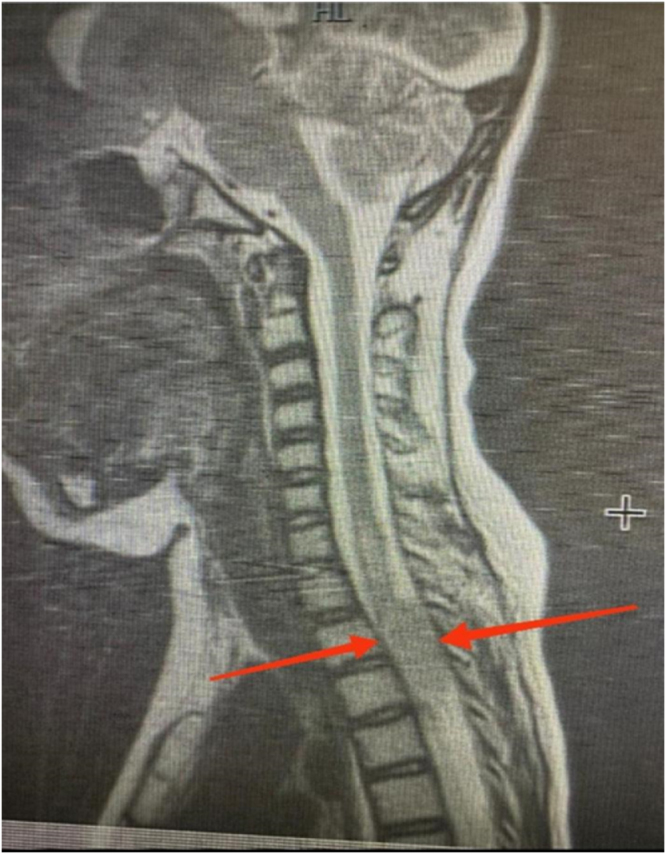
Sagittal T2-weighted MRI of the cervicothoracic spine showing a posterior epidural mass at C7–T1 compressing the spinal cord, consistent with drop metastasis.

**Figure 6.**
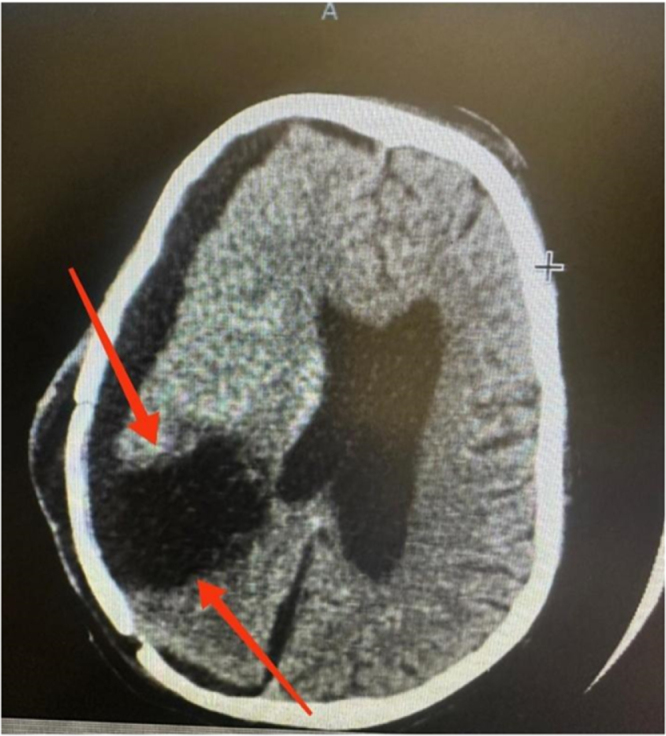
Postoperative non-contrast CT brain showing a well-defined pseudomeningocele at the craniotomy site.

According to symptoms, an MRI of the dorsal spine was performed, which showed a lobulated posterior epidural mass at the cervicothoracic junction (≈C7–T1) producing dorsal CSF effacement and anterior compression of the spinal cord. The presence of this mass was suspicious for neuraxial (drop) metastasis of STF/HPC (as shown in Fig. 5). A CT scan of the brain was performed (Fig. 6), and repeat surgical excision was advised. However, due to financial limitations, the family declined further surgical or oncological intervention.

### Follow-up

Due to socioeconomic constraints, the patient was unable to undergo further surgical intervention, radiotherapy, or oncologic evaluation. She was discharged with symptomatic management. Subsequent follow-up was limited; however, at last contact, she continued to have visual impairment and intermittent lower-limb weakness. Long-term outcomes remain unknown.

## Discussion

This case provides several clinically meaningful insights that are not emphasized in previously published pediatric reports. First, the unusual early spinal drop metastasis occurring within weeks of gross total resection. Second, the coexistence of early postoperative pseudomeningocele and acute neurological deterioration raises the possibility that subtle postoperative complications can mask or overlap with signs of metastatic spread. Lastly, the diagnostic and therapeutic course in a resource-constrained setting highlights real-world limitations that can delay lifesaving interventions.

What sets this case apart from previous pediatric reports is the rapid onset of spinal drop metastasis. Our case has the shortest time interval for the drop metastasis so far. Normally, the time interval between the initial diagnosis and spinal metastasis is 9.5 years on average^[^10^]^. Published pediatric evidence of spinal (drop) metastasis from intracranial SFT/HPC is extremely limited. We found one clearly documented pediatric case of intradural spinal dissemination (Uçpınar *et al*, Neurology-Asia 2021), in which spinal metastasis appeared ~25 months after the cranial diagnosis. Several other pediatric intracranial SFT/HPC reports exist but do not report spinal dissemination on follow-up. In contrast, the adult literature describes many examples of spinal metastasis with a wide latency range (months to >10 years), as shown in Table 2.

**Table 2:** Comparison of this case with literature.

Source	Age	WHO grade	Interval till spinal metastasis occurrence	Spinal site
Uçpınar *et al*^[^29^]^	6 years	Grade III	left25 months (2 years)	Intradural
Hayenga *et al*^[^30^]^	Adult (case series)	Grade III	Examples in literature	Thoracic/lumbar intradural/extradural examples
			This report reviews multiyear latency	
Joo *et al*^[^31^]^	45	–	13 years	L2 vertebral body
Ali *et al*^[^10^]^	53	–	Literature Review many cases with multiyear intervals	Thoracic and lumbar spine
			Shows range	
Sweid *et al*^[^32^]^	Adult	–	9 years (case narrative)	Spinal metastasis documented
Qian *et al*^[^33^]^	47	Grade I	About 1 year	Lumbar spine

Intracranial SFT/HPC is a very rare entity in pediatric neurosurgery. Population-based epidemiological studies have revealed that meningeal SFT/HPCs are less than 1% of all primary central nervous system tumors and even less frequent in children. Only 220 cases have been reported during 1996–2011, and one-fifth were intracranial^[^11,12^]^. The rarity of this diagnosis in childhood is highlighted by the fact that most of the literature consists of isolated case reports and not in organized series^[^5^]^. Our case adds to the infrequent pediatric literature, since intracranial SFT/HPC in an 11-year-old is a rare occurrence worthy of complete documentation. Our case is among the few pediatric patients with early postoperative spinal drop metastasis, which highlights the importance of close long-term follow-up and treatment planning specific to each patient.

In the child, intracranial SFT/HPC is unheard of, to the extent of being virtually unknown, with only a few cases described up to now. The world literature cites only >30 cases of pediatric SFT between 1998 and 2017^[^5^]^. These findings corroborate the wide age range but extremely low incidence of SFT/HPC in the childhood CNS.

The histopathological features seen in our case, such as the typical “staghorn” vascular pattern, marked cellularity, and increased mitotic activity, are characteristic of Grade III SFT/HPC based on current WHO classification criteria^[^13^]^. The immunohistochemical pattern, especially the nuclear expression of STAT6, is a good surrogate marker for the pathognomonic NAB2–STAT6 fusion gene characteristic of this tumor type^[^14,15^]^. This molecular transformation, first described in 2013, has revolutionized the diagnosis and description of SFT/HPC, providing a unifying genetic etiology for heretofore considered disparate entities^[^15,16^]^. Our patient’s elevated Ki-67 labelling index is particularly concerning in pediatric patients since it carries increased risk of recurrence and metastatic potential^[^17^]^.

Extracranial metastasis is common to the lungs, liver, and bone^[^7^]^. Drop metastases in the spine are rare and usually occur years after the first treatment^[^8,18^]^. There are reports that dissemination occurs through seeding by CSF, resulting in intradural or epidural deposits, occasionally including the cauda equina^[^19^]^. Our case developed early cervicothoracic metastasis in weeks after primary surgery, showing the potential aggressiveness of high-grade pediatric tumors.

This has also been well reported in medulloepitheliomas and high-grade gliomas, but is extraordinarily uncommon within SFT/HPC^[^20^]^. Evidence-based treatment approaches are limited in number, and no definite surveillance standard is available; management is thus often analogically extrapolated from other soft tissue sarcoma subtypes^[^19^]^. These spinal drop metastases have catastrophic implications for prognosis and outcome.

Due to the rarity of pediatric SFT/HPC, treatment relies on analogous principles as for adults, and the maximal safe resection is the treatment cornerstone^[^5^]^. Gross total resection, whenever feasible, provides the best long-term outcomes and decreases the risk of local recurrence in comparison to subtotal resection^[^21^]^. In our practice, the complete resection of the tumor as a gross total was possible with the aid of neuronavigation-guided techniques, particularly helpful in children, where preserving eloquent areas of the brain is most critical.

Adjuvant radiotherapy for childhood SFT/HPC is controversial due to concern about long-term neurocognitive impairment in the developing brain^[^22^]^. Modern radiation techniques, including intensity modulated radiotherapy and proton beam therapy, may decrease long-term sequelae but provide adequate tumor control^[^23,24^]^. Chemotherapy has been unsuccessful, but there is evidence towards the use of antiangiogenic agents such as bevacizumab in resistant disease^[^25^]^.

The development of pseudomeningocele in our patient is an established complication following craniotomy. The development of pseudomeningocele in just 5.1% of 1648 patients, according to a retrospective study, only indicates that it is a rare complication^[^26^]^. Pseudomeningocele formation is due to the accumulation of CSF within the subcutaneous space due to defects in the dura or incompetent closure of the dura. Postoperative pseudomeningocele is very rare and very rarely described in the literature. It has no guidelines for management^[^27^]^. The pseudomeningocele may also cause neurological deficits, as in our case^[^28^]^.

In conclusion, clinicians treating pediatric SFT/HPC should consider early postoperative MRI of the entire spine, ideally within 2–4 weeks after cranial surgery, as well as short-interval follow-up scans (every 3–6 months) in high-grade tumors. Multidisciplinary decision making (neurosurgery, oncology, radiation oncology, and pathology) should be done due to the absence of pediatric guidelines. These steps can cause earlier detection of metastatic or recurrent disease and improve clinical decision making.

## Conclusion

Pediatric intracranial SFT/HPC is very rare and sometimes exhibits aggressive biological behavior. Our report addresses the rare combination of early postoperative pseudomeningocele formation followed by rapid cervicothoracic drop metastasis in an 11-year-old girl. These results highlight the need to maintain a low threshold for early detection of metastatic spread when new neurologic signs are present, even in the early postoperative period. The unusually rapid onset of drop metastasis in this child expands the known biological spectrum of pediatric SFT/HPC and emphasizes that early postoperative recurrence should be considered even after visually complete resection.

With a lack of evidence-based treatment guidelines in children, management must be individualized, incorporating maximal safe resection, consideration of adjuvant modalities, and multidisciplinary follow-up.

## Patient perspective

The patient’s family expressed initial relief after gross total resection and expected improvement in seizures and vision. However, the rapid postoperative decline and discovery of spinal metastasis caused significant emotional distress. The family reported that financial limitations were the main reason for declining further surgery and oncology care. They hoped that reporting their child’s case might help raise awareness about rare pediatric brain tumors and challenges faced in resource-limited settings.

## Data Availability

Data are available on request from the authors.
